# Efficacy of dehydroepiandrosterone to improve ovarian response in women with diminished ovarian reserve: a meta-analysis

**DOI:** 10.1186/1477-7827-11-44

**Published:** 2013-05-16

**Authors:** Amarin Narkwichean, Walid Maalouf, Bruce K Campbell, Kannamannadiar Jayaprakasan

**Affiliations:** 1 Division of Obstetrics and Gynaecology, School of Clinical Sciences, University of Nottingham, Nottingham, UK; 2 Faculty of Medicine, Srinakharinwirot University, Bangkok, Thailand; 3 Reproductive Medicine and Fertility Unit Lead, Royal Derby Hospital, Derby, UK

**Keywords:** Dehydroepiandrosterone (DHEA), Diminished ovarian reserve (DOR), Poor ovarian response, *In vitro* fertilization (IVF)

## Abstract

Women with diminished ovarian reserve often respond poorly to controlled ovarian stimulation resulting in retrieval of fewer oocytes and reduced pregnancy rates. It has been proposed that pre-IVF Dehydroepiandrosterone (DHEA) adjuvant therapy may improve ovarian response and pregnancy rates in women with diminished ovarian reserve. This meta-analysis aims to investigate efficacy of DHEA as an adjuvant to improve ovarian response and IVF outcome in women with diminished ovarian reserve. Electronic databases were searched under the following terms: (DHEA) and (diminished ovarian reserve) and/or (poor response). Studies were included if they reported at least one of the following outcomes; clinical pregnancy rate, number of oocytes retrieved, miscarriage rate. We identified 22 publications determining effects of DHEA in clinical trials. Only 3 controlled studies were eligible for meta-analysis. There was no significant difference in the clinical pregnancy rate and miscarriage rates between women pre-treated with DHEA compared to those without DHEA pre-treatment (RR 1.87, 95% CI 0.96-3.64; and RR 0.59, 95% CI 0.21-1.65, respectively). The number of oocytes retrieved (WMD -1.88, 95% CI -2.08, 1.67; P < 0.001) was significantly lower in the DHEA group. In conclusion, based on the limited available evidence from a total of approximately 200 IVF cycles, there are insufficient data to support a beneficial role of DHEA as an adjuvant to controlled ovarian stimulation in IVF cycle. Well-designed, randomised controlled trials as well as more exact knowledge about DHEA mechanisms of action are needed to support use of DHEA in standard practice for poor-responders.

## Background

Ovarian ageing, as manifested by reduced ovarian reserve, is responsible for the well-established observation of age related decline in fertility [1,2] and of age related increase in adverse reproductive events such as miscarriages [3] and aneuploid pregnancies [4]. While ovarian ageing is one of the major determinants of outcome following in-vitro fertilization (IVF), women with reduced ovarian reserve often respond poorly to controlled ovarian stimulation resulting in retrieval of fewer oocytes, producing poorer quality embryos and reduced implantation rates and pregnancy rates. Incidence of poor ovarian response, a measure of reduced ovarian reserve, ranges from 9-24% in the earlier report [5] but is increasing [6] because of global rise in the number of women who defer conceiving to their 30s or 40s. Various treatment regimens including different stimulation protocols and adjuvant therapies have been reported to improve ovarian response and pregnancy rates in women with diminished ovarian reserve but none of them have been proven to be superior over the others to recommend any one of them as the best protocol of choice [7].

It has been proposed that oral administration of Dehydroepiandrosterone (DHEA), an adrenal androgen, may have anti-ageing effects and may improve ovarian response and pregnancy rates in women with reduced ovarian reserve during IVF. Casson et al., in the year 2000, who had interest in the use of DHEA for hormone replacement therapy (HRT) were the first group that reported the benefits of DHEA supplementation for improving the response to ovarian stimulation in a case series [8]. Since then, a few controlled studies including a randomised controlled study, but with small sample sizes, have subsequently reported benefits of DHEA supplementation to improve ovarian response and IVF outcome [9]. While the mechanism of action for this improved IVF outcome following DHEA administration remains speculative, controlled studies have demonstrated oral DHEA supplementation increases serum IGF-I concentrations, which are known to have a positive effect on follicular development and oocyte quality. Further, it is well established that androgens can directly influence ovarian follicle development by local intra-ovarian androgen receptor mediated actions [10,11] and it is therefore possible that DHEA may enhance the follicular environment through: augmentation of the growth promoting and survival enhancing effect of IGF-I; LH-stimulated follicular androgen and oestrogen production [12]; and the augmentation of granulosa cell FSH-receptor (FSH-r) expression and associated increase in the number of growing preantral and small antral follicles [13,14]. In addition, given the published report of DHEA on reducing miscarriage rates in older patients, a direct- or indirect- effect of DHEA at the level of the oocyte cannot be ruled out. DHEA could potentially improve oocyte quality via the GH axis through the promotion of DNA repair in oocytes [15]. An effect of DHEA on mitochondrial activity in both follicular cells and oocytes is also possible since androgens have been shown to beneficially affect mitochondrial function [16].

A recent world-wide survey has shown that over a quarter (26%) of IVF clinicians add DHEA as an adjuvant to IVF treatment protocols in women with poor ovarian response [6]. Despite widespread use of DHEA, clinical evidence as well as knowledge regarding underlying mechanisms of DHEA on improvement of ovarian response is still limited. A recent systematic review by Sunkara et al. investigating the role of androgens (testosterone, DHEA, and aromatase inhibitor- Letrozole) in poor-responders undergoing IVF treatment could not show significantly improvement in terms of pregnancy rates and other parameters such as number of oocytes retrieved [17]. While Sunkara et al.’s review included all the androgen adjuvants, there is no systematic review and meta-analysis reported specifically on the role of DHEA alone in women with diminished ovarian reserve. This systematic review aims to summarize the role of DHEA as an adjuvant to stimulation protocol in women with diminished ovarian reserve or poor-responders based on meta-analysis of the published controlled studies.

## Methods

We searched EMBASE (1980 to December 2012), MEDLINE (1948 to December 2012), Pubmed and Cochrane Library for all relevant articles under the following Medical Subject Headings (MeSH) terms to generate subsets of studies; i) ‘DHEA’ or Dehydroepiandrosterone’, ii) ‘Poor response’ or ‘low response’, and iii) ‘Diminished ovarian reserve’ or ‘Premature ovarian aging’. Combining these subsets together (subset i with either ii or iii) by ‘AND’ to identify citations appropriate to the question ‘The effect of DHEA supplementation on ovarian stimulation outcomes in diminished ovarian reserve and/or poor responses patients’. The search also tracked on ISI conference abstracts as well as on-going randomised controlled trials registered on ISRCTN database. In addition, all primary papers’ bibliographies were explored to recognize cited publications which had not been identified by electronic-based searches. Only articles written in English were included in the meta-analysis. The searches were conducted by two reviewers independently (AN and KJ).

The target population was either poor-responders or those with diminished ovarian reserve, as described above, who were undergoing ovarian stimulation plus IVF/ICSI. DHEA was supplemented before ovarian stimulation in the study group while neither was used in the control. The primary outcome was the clinical pregnancy rate and the secondary outcomes were oocyte yield (numbers of oocytes retrieved), miscarriage rate, live birth rate and aneuploidy rates.

All full manuscripts were reviewed for the selection and exclusion of publications with predefined inclusion criteria by two reviewers (AN and KJ) independently. Extraction data for each study, e.g. study design, inclusion/exclusion criteria, population characteristics, definition of diminished ovarian reserve, stimulation protocol, and outcomes, was separately done by two of the authors (AN and KJ) using pre-determined tables and form. Disagreements about either article selection or data extraction were resolved by consensus or arbitration by a third reviewer (BKC). The Newcastle-Ottawa Scale (NOS) scoring system [18] was used to assess methodology and quality of observational studies (non-randomised trials). For RCT, the full publication was scrutinized to identify study characteristics; randomisation, allocation, blinding, and intention-to-treat analysis.

### Statistical analysis

Relative risks (RR) from individual studies were analysed using random effects models [19]. Heterogeneity of exposure effects was evaluated graphically using forest plots (Lewis and Clarke, 2001) and statistically using the I^2^ statistic to quantify heterogeneity across studies [20]. Statistical analyses were performed using RevMan 5.1 software (Cochrane Collaboration, Oxford, UK).

## Results

The search yielded 38 publications of which 16 excluded by screening through titles and abstracts, including one paper that is not published in English. Full manuscripts were retrieved for the remaining 22 articles, which included 3 case series [21-23], 3 case-control [24-26], 7 prospective self-controlled studies [8,12,27-31], 1 randomised controlled trial (RCT) [9], 3 abstract oral/poster presentations [32-34], and 5 reviews [22,35-38]. Studies that have matched controls were identified eligible for analyses. Study characteristics of controlled studies are presented in Table 1 whereas quality of all studies included in the meta-analysis is shown in Table 2.

**Table 1:** Study characteristics of controlled studies (both RCT and non-RCTs) of DHEA supplementation in poor-responders or diminished ovarian reserve

**Articles**	**Study design**	**Inclusion criteria**	**Cases/ Controls**	**Intervention (DHEA doses and duration)**	**Stimulation protocol**	**Embryo transfer**	**Outcomes**	**Notes**
Barad D, et al. (2007)	Case–control	**POA** defined by age-specific baseline FSH levels > 95% CI of mean value for the age group; but < 12 mIU/ml **DOR** defined as baseline FSH > 12 mIU/ml and/or estradiol level ≥ 75 pg/ml	89 cases* and 101 controls *only 64 of 89 undergoing IVF	Cases : DHEA 25 mg three times daily for mean duration 73 days continuously until	-Allow cases to conceive naturally; the other entered IVF using microdose agonist	Day 3 embryo transfer	-Clinical pregnancy rate -No. of retrieved oocytes -Implantation rate -Miscarriage rate -Normal day 3 embryos -Time from initial visit to pregnancy (Cox regression analysis)	-Cases were slightly older (P < 0.05) -Fertility treatments were different (P < 0.001) -Women in control entered IVF cycle more rapidly
				positive pregnancy test	flare followed by high dose FSH + HMG (300–450 + 150 IU)			
				Control : None	-Similar protocol for both cases/controls			
Wiser A, et al. (2010)	RCT (open- labeled)	Age ≤ 41 yr, Poor response, previous IVF cycle with high dose Gn (FSH 300 IU) with oocyte <5 or cycle cancellation	17 Cases	Cases : DHEA 75 mg/day orally ≥ 6 weeks before stimulation	- Similar protocol for both cases/controls	Day 2–3 embryo transfer	-Peak estradiol levels	Counted 55 IVF from 33 patients (both arms went through
			16 Controls	Control : None	- Standard long GnRH agonist protocol	Up to 3 embryos	-No. of retrieved oocytes	Including of repeat cycles without adjustment of randomisation
					- Using rFSH 450 IU + rLH 150 IU		-Embryo quality and No. of reserve embryo	
Gleicher N, et al. (2010)	Case–control	DOR defined by abnormally age specific hormone levels deviated from 95% CI; elevated FSH or low AMH	22 Cases	Cases : DHEA 25 mg three times daily At least 4 weeks before stimulation	Microdose agonist flare followed by high dose FSH + HMG (300–450 + 150 IU)	Not being stated	-Pregnancy and live birth rates (secondary outcome)	Clinical pregnancy rate, miscarriage and No. of oocyte retrieved (our outcomes) are not the main outcome of the study.
			44 matched Controls (1st single IVF cycle analysis only)		-Similar protocol for both cases/controls	Pregnancy was not outcome of interest	-Aneuploidy rate	
				Control : None			-No. of oocytes retrieved	
							-Total gonadotropin dosage	

*POA: Premature Ovarian Aging, DOR: Diminished Ovarian Reserve.

**Table 2:** **Quality of controlled studies passing eligibility criteria presented by stratification of research methodology and Newcastle-Ottawa scale**^**a **^**(for non-randomised observational studies)**

**Author/Year**	**Design**	**Randomisation**	**Blinding**	**Sample size estimation**	**Analysis**	**Newcastle-Ottawa scale**		
						**Selection**	**Comparability**	**Outcome**
Barad D, et al. (2007)	Case -control study	None	None	N/A	Intention to treat analysis	***	*	**
Wiser A, et al. (2010)	Randomised controlled study	-Computer generated random numbers	None	Not done	Intention to treat analysis			
		- allocation concealment by sealed envelope			(No-drop out)			
Gleicher N, et al. (2010)	Case -control study	None	None	N/A	Intention to treat analysis	***	*	***

^a^ Wells GA et al. The Newcastle-Ottawa Scale (NOS) for assessing the quality if nonrandomized studies in meta-analysis, available from: URL: http://www.ohri.ca/programs/clinical_epidemiology/oxford.asp [cited 2012 June 1].

### Primary outcome: clinical pregnancy rate

**Figure 1:**
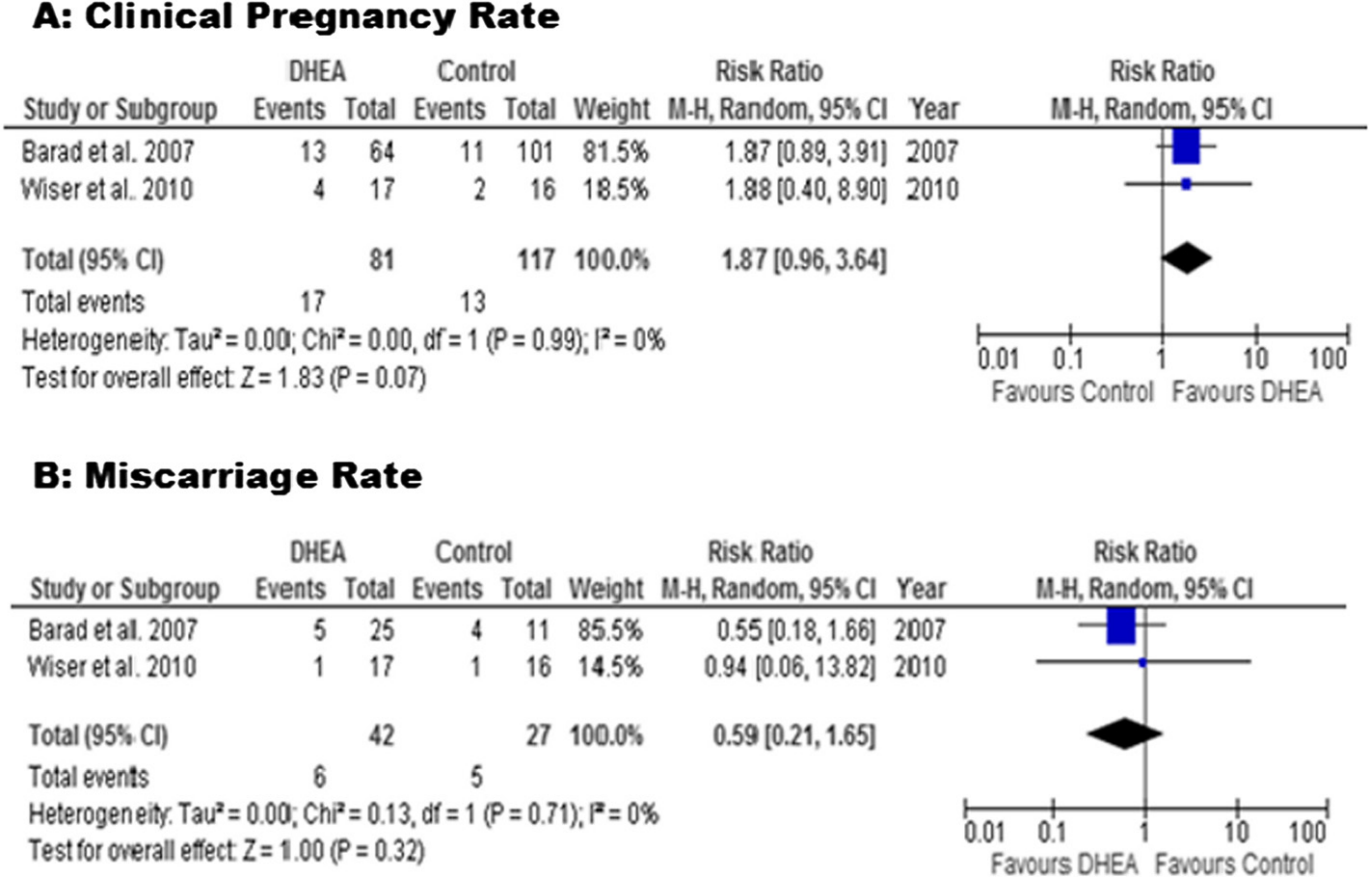
**Meta-analysis of clinical pregnancy and miscarriage rates.** Meta-analysis of studies of DHEA supplementation versus controls for outcome of **A**) clinical pregnancy rates and **B**) Miscarriage rates in DOR or poor responders undergoing IVF cycle.

Only two studies, one RCT [9] and one non-RCT [25], were selected for meta-analysis for the clinical pregnancy rate (CPR) outcome. Due to the fact that Wiser’s study reported cumulative pregnancy outcome over two consecutive IVF cycles, only first cycle data was taken into account. Pooling data together (Figure 1A), there was no significant difference in the clinical pregnancy rate between women pre-treated with DHEA compared to those without DHEA pre-treatment (RR 1.87, 95% CI 0.96, 3.64; P=0.07). While there was homogeneity observed between the two included studies (I^2^< 1%), the study designs were completely different as one was a case control study and the other, a randomised controlled trial.

### Secondary outcome: miscarriage rate

Similar to primary outcome, only two published controlled studies were eligible for analysis [9,25]. The results from these studies showed that there was no difference between the DHEA and control groups (RR 0.59, 95% CI 0.21, 1.65; Figure 1B).

Further literature review revealed one particularly retrospective study from Gleicher et al. (2009) which specifically examines miscarriage rates. In this study, they reported that DHEA supplemented pregnancies in women with diminished ovarian reserve had lower miscarriage rates when compared to the national United States IVF database (OR 0.49; P=0.04) [26]. Because of lack of comparability between cases and controls, who did have diminished ovarian reserve and who did not, this study was therefore, excluded from meta-analysis.

### Oocytes retrieved

**Figure 2:**
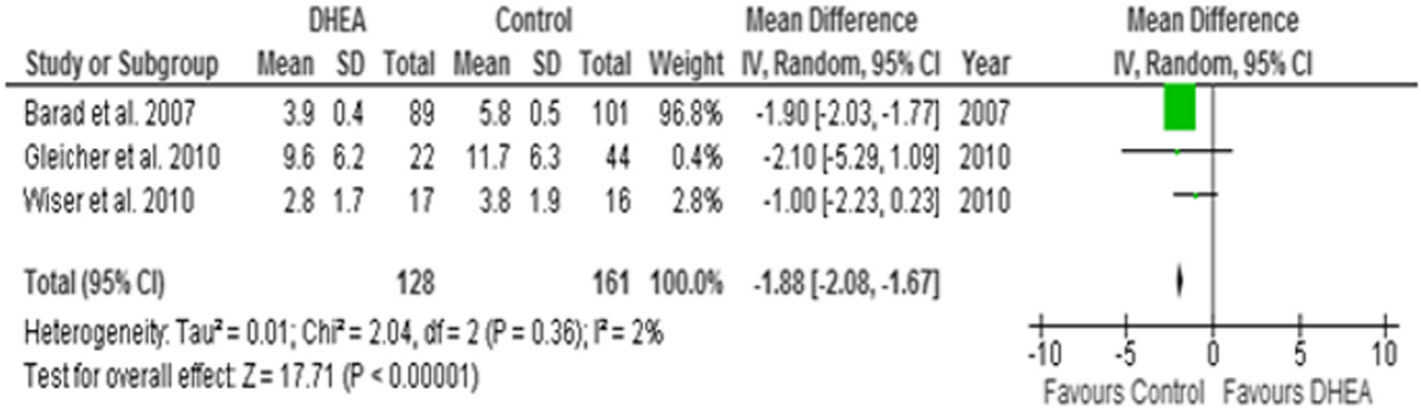
**Meta-analysis of numbers of oocytes.** Meta-analysis of studies of DHEA supplementation versus controls for outcome of numbers of oocytes retrieved in DOR or Poor responders undergoing IVF cycle.

Regarding number of oocytes, meta-analysis of the three studies, one RCT [9] and two non-RCT [24,25], demonstrated a significantly lower number of oocytes retrieved in DHEA treated women when compared to the controls (WMD -1.88, 95% CI -2.08, -1.67). However, a significant heterogeneity of 74% (I^2^) among studies was observed (Figure 2).

## Discussion

This systematic review of the controlled studies that reported the effect of pre-treatment DHEA on IVF outcome in women with diminished ovarian reserve suggests that DHEA does not improve the quantitative ovarian response and pregnancy outcome. While the ovarian response as defined by the number of oocytes retrieved was significantly lower, the clinical pregnancy rate was marginally superior with a relative risk of 1.87 (95% CI 0.96-3.64; P=0.07) in the DHEA group. The miscarriage rate was similar between the DHEA and control groups on meta-analysis of the two reported controlled studies. This finding, however, is based on few data as there was only one study [9] which reported live birth rate, which was similar between the DHEA and control groups when only one cycle per participant was considered for analysis.

While there are several self-controlled case series on reported significant benefits in terms of ovarian response and pregnancy rates with the use of pre-treatment DHEA adjuvant during IVF, this systematic review of controlled studies failed to demonstrate such a benefit. However, while noting the trend of a positive effect of DHEA on the pregnancy rate in this review, the lack of a significant difference may be because of a small sample size with the overall number of participants that are included in the meta-analysis is only 198. In the study of Barad et al. 2007 included in this review, the authors have also reported spontaneous pregnancy (n=6/16) and pregnancy following IUI (6/9), which occurred during the three to four months waiting time of pre-IVF DHEA adjuvant treatment [25]. When these data were included in the meta-analysis, there was a significantly increased pregnancy rates in the DHEA arm over the controls (RR 2.46 95% CI 1.35, 4.48; P=0.003). Since our objective was to investigate the effect of DHEA in IVF cycle, we have included only the IVF population in the primary analysis.

At present, there is only a single small randomised controlled trial by Wiser et al. 2010 reported in literature [9]. The small sample size in this study resulted in only a minimal effect toward the result of the meta-analysis (Figures 1 and 2). Furthermore, many limitations and weaknesses of Wiser’s study have been criticised. First, there was no priori sample size estimation in the study. The authors included two cycles with varying duration of DHEA adjuvant treatment (7 – 18 weeks) and the authors continued the trial until a significant difference in cumulative live birth rate between the study and control groups was achieved. There was no difference in the live birth rate between the study and control groups following their first cycle of IVF with the mean duration of DHEA therapy in the study group was for only about 8.5 weeks. Secondly, both patients and health care providers were not blinded in this study therefore bias could occur, or patients in the control group might have sought over-the-counter medication for themselves. And finally, one letter to the editor expressed concern over the statistical analysis in this study suggesting that because the author had included two consecutive IVF cycle data from each group, Kaplan-Meier survival analysis should have been used rather than Fisher’s exact test, which was originally reported [39].

Most data on the favourable effect of DHEA adjuvant supplementation currently come from the study group led by Barad and Gleicher at the Centre of Human Reproduction, New York. They have published a series of self-controlled studies and retrospective case control analyses in which the benefits of DHEA are summarized as follows; i) increased oocyte yield [12] ii) higher fertilization rate [12] iii) improved embryo morphological grading [12] iv) increased pregnancy rate [25], v) lower miscarriage rate when compared to the national IVF statistics [26], and vi) lower aneuploidy rate [24]. We have included two of their suitable controlled studies in our meta-analysis (Table 1). The authors also suggested that the benefit of DHEA treatment would be most effective if it is supplemented for at least 3-4 months, which is equal to the time needed for the early growing follicles to reach the gonadotropin-responsive stage [35]. Therefore, they believed that DHEA acts in both ovarian recruitment and early folliculogenesis. If this hypothesis is true, the duration of DHEA treatment is possibly a key factor in effecting a favourable change in ovarian response and pregnancy rates following IVF.

The data from the self-controlled studies should be interpreted with caution because of potential bias. In one review by Urman and Yakin, the authors retrospectively analysed IVF outcome in the second cycle of 801 patients who have history of poor ovarian response (production of ≤ 4 oocytes) in the first IVF cycle treatment in their centre. It was found that almost 40% of this cohort developed better response yielding more than 4 oocytes in their next cycle [37]. These data also confirm that poor response cannot be predicted only by history. Using other predictive tools to determine ovarian reserve, for instance antral follicle count or serum AMH, or both, is also critical to identify patients who will mostly benefits from DHEA in the clinical trial [40].

This systematic review did not include any self controlled studies, but included only the studies that had a group of contemporaneous controls. Our results in this review indicate that DHEA decreases the ovarian response as indicated by reduced number of oocytes retrieved at egg collection in the study arm. While this finding is surprising and it is difficult to come up with a scientific explanation, this systematic review is limited by a small number of treatment cycles included in the meta-analysis and by the heterogeneity of the included studies. On the contrary to the above finding, as indicated by trends of improving clinical pregnancy and of reducing miscarriage albeit no statistical significance, DHEA may have a positive effect on improving oocyte and embryo quality. It is already established that poor oocyte quality, which leads to producing poorer quality embryos, represents one of the clinical presentations of ovarian ageing [41]. The finding of potential effects of DHEA on oocyte quality and ovarian response warrant well-designed randomised controlled clinical trials of an adequate sample size using well-defined uniform inclusion criteria before recommending the use of DHEA in standard assisted reproduction treatment. In addition, further in-vivo and in-vitro embryological and endocrinological research to elucidate the mechanism of action of DHEA on ovarian folliculogenesis and on oocyte/embryo quality are also required.

Recent meta-analysis which evaluated the effect of adjuvant androgens (DHEA or Testosterone) or androgen-modulating agents (Letrozole, aromatase inhibitor) in previous poor responders has failed to demonstrate any significant difference in the ongoing pregnancy rate, live birth rates, and numbers of oocytes retrieved when compared with the control group, who have had no adjuvant therapy [17]. Another systematic review by Bosdou et al. 2012 reports a significant increase in clinical pregnancy and live birth rates in poor responder women who are pre-treated with transdermal testosterone, but not in other androgen modulating agents including DHEA, when compared to controls [42]. The review by Bosdou et al. on DHEA included only one study with a small sample size (n=33) in contrast to this review in which we included two other eligible controlled studies with larger sample sizes. However, it is important to note that all the reviews including ours are still limited by small sample sizes and heterogeneity between the studies included. In addition, there is a wide methodological variation in different studies in terms of different preparations that are used as adjuvants, and duration and timing, in relation to down regulation and ovarian stimulation during IVF, of adjuvant treatments. This conflicting data regarding the use of androgen and androgen modulating agents require further investigation by well-designed randomised controlled trials.

## Conclusions

In conclusion, our systematic review of the controlled studies on the effect of pre-treatment DHEA on IVF outcome in women with diminished ovarian reserve suggests that DHEA does not improve the quantitative ovarian response and pregnancy outcome. Based on these data, DHEA adjuvant therapy cannot be recommended in diminished ovarian reserve for improving IVF outcome. However, as the sample size in this analysis was small and the effect of DHEA on pregnancy rates approached close to statistical significance, further large scale multicentre randomised controlled studies are required to clarify the benefits of DHEA adjuvant therapy in routine clinical management of predicted poor responders.

## Competing interests

The authors declare that they have no competing interests.

## Authors’ contributions

AN and KJ were responsible for the conception, design, literature identification/selection, data extraction, analysis and interpretation. Critical evaluation and resolving disagreements regarding selection, data extraction and analysis were done by BKC. All four authors (AN, WM, BKC, KJ) were responsible for writing the manuscript, revision of the article and statistical analyses. All authors read and approved the final manuscript.

## Authors’ information

Amarin Narkwichean, M.D., M.Med.Sci., RTCOG, Currently a PhD student at the Division of Obstetrics and Gynaecology, School of Clinical Sciences, University of Nottingham, United Kingdom & Clinical Lecturer, Faculty of Medicine, Srinakharinwirot University, Thailand. Walid Maalouf PhD, Lecturer in Embryology, Division of Obstetrics and Gynaecology, School of Clinical Sciences, University of Nottingham, United Kingdom. Bruce Campbell PhD., DSc., Professor, Chair of Reproductive Physiology, Division of Obstetrics and Gynaecology, School of Clinical Sciences, University of Nottingham, United Kingdom. Kannamannadiar Jayaprakasan, M.D., PhD., MRCOG., Honorary Associate Professor, Division of Obstetrics and Gynaecology, School of Clinical Sciences, University of Nottingham, United Kingdom. Subspecialist in Reproductive Medicine and Fertility Unit Lead, Royal Derby Hospital, Derby.

## References

[B1] Menken J Trussell J Larsen U Age and infertility Science 1986 233 1389 1394 10.1126/science.3755843 3755843

[B2] Templeton A Morris JK Parslow W Factors that affect outcome of in-vitro fertilisation treatment Lancet 1996 348 1402 1406 10.1016/S0140-6736(96)05291-9 8937279

[B3] Trout SW Seifer DB Do women with unexplained recurrent pregnancy loss have higher day 3 serum FSH and estradiol values? Fertil Steril 2000 74 335 337 10.1016/S0015-0282(00)00625-7 10927054

[B4] Freeman SB Yang Q Allran K Taft LF Sherman SL Women with a reduced ovarian complement may have an increased risk for a child with Down syndrome Am J Hum Genet 2000 66 1680 1683 10.1086/302907 10733467 PMC1378004

[B5] Keay SD Liversedge NH Mathur RS Jenkins JM Assisted conception following poor ovarian response to gonadotrophin stimulation Br J Obstet Gynaecol 1997 104 521 527 10.1111/j.1471-0528.1997.tb11525.x 9166190

[B6] Leong M Patrizio P Poor responders: how to define, diagnose and treat? 2010 http://www.ivf-worldwide.com/survey/poor-responders/results-poor-responders.html. 10.1016/j.rbmo.2015.03.002 25892496

[B7] Pandian Z McTavish AR Aucott L Hamilton MP Bhattacharya S Interventions for 'poor responders' to controlled ovarian hyper stimulation (COH) in in-vitro fertilisation (IVF) Cochrane Database Syst Rev 2010 20 CD004379 10.1002/14651858.CD004379.pub3 PMC13243861 20091563

[B8] Casson PR Lindsay MS Pisarska MD Carson SA Buster JE Dehydroepiandrosterone supplementation augments ovarian stimulation in poor responders: a case series Hum Reprod 2000 15 2129 2132 10.1093/humrep/15.10.2129 11006185

[B9] Wiser A Gonen O Ghetler Y Shavit T Berkovitz A Shulman A Addition of dehydroepiandrosterone (DHEA) for poor-responder patients before and during IVF treatment improves the pregnancy rate: a randomized prospective study Hum Reprod 2010 25 2496 2500 10.1093/humrep/deq220 20729538

[B10] Hillier SG Tetsuka M Role of androgens in follicle maturation and atresia Baillieres Clin Obstet Gynaecol 1997 11 249 260 10.1016/S0950-3552(97)80036-3 9536210

[B11] Walters KA Simanainen U Handelsman DJ Molecular insights into androgen actions in male and female reproductive function from androgen receptor knockout models Hum Reprod Update 2010 16 543 558 10.1093/humupd/dmq003 20231167

[B12] Barad D Gleicher N Effect of dehydroepiandrosterone on oocyte and embryo yields, embryo grade and cell number in IVF Hum Reprod 2006 21 2845 2849 10.1093/humrep/del254 16997936

[B13] Nielsen ME Rasmussen IA Kristensen SG Christensen ST Møllgård K Wreford Andersen E Byskov AG Yding Andersen C In human granulosa cells from small antral follicles, androgen receptor mRNA and androgen levels in follicular fluid correlate with FSH receptor mRNA Mol Hum Reprod 2011 17 63 70 10.1093/molehr/gaq073 20843821

[B14] Walters KA Allan CM Handelsman DJ Androgen actions and the ovary Biol Reprod 2008 78 380 389 10.1095/biolreprod.107.064089 18003945

[B15] Menezo Y Dale B Cohen M DNA damage and repair in human oocytes and embryos: a review Zygote 2010 18 357 365 10.1017/S0967199410000286 20663262

[B16] Pitteloud N Mootha VK Dwyer AA Hardin M Lee H Eriksson KF Tripathy D Yialamas M Groop L Elahi D Hayes FJ Relationship between testosterone levels, insulin sensitivity, and mitochondrial function in men Diabetes Care 2005 28 1636 1642 10.2337/diacare.28.7.1636 15983313

[B17] Sunkara SK Pundir J Khalaf Y Effect of androgen supplementation or modulation on ovarian stimulation outcome in poor responders: a meta-analysis Reprod Biomed Online 2011 22 545 555 10.1016/j.rbmo.2011.01.015 21493151

[B18] Wells GA Shea B O'connell D Peterson J Welch V Losos MPT The Newcastle-Ottawa Scale (NOS) for assessing thequality if nonrandomized studies in meta-analyses 2004 Ottawa http://www.ohri.ca/programs/clinical_epidemiology/oxford.asp

[B19] DerSimonian R Laird N Meta-analysis in clinical trials Control Clin Trials 1986 7 177 188 10.1016/0197-2456(86)90046-2 3802833

[B20] Higgins JP Thompson SG Quantifying heterogeneity in a meta-analysis Stat Med 2002 21 1539 1558 10.1002/sim.1186 12111919

[B21] Barad DH Gleicher N Increased oocyte production after treatment with dehydroepiandrosterone Fertil Steril 2005 84 756 16169414 10.1016/j.fertnstert.2005.02.049

[B22] Mamas L Mamas E Dehydroepiandrosterone supplementation in assisted reproduction: rationale and results Curr Opin Obstet Gynecol 2009 21 306 308 10.1097/GCO.0b013e32832e0785 19610174

[B23] Sönmezer M Cil AP Oktay K Ongoing pregnancies from early retrieval of prematurely developing antral follicles after DHEA supplementation Reprod Biomed Online 2009 19 816 819 10.1016/j.rbmo.2009.09.025 20031022

[B24] Gleicher N Weghofer A Barad DH Dehydroepiandrosterone (DHEA) reduces embryo aneuploidy: direct evidence from preimplantation genetic screening (PGS) Reprod Biol Endocrinol 2010 8 140 10.1186/1477-7827-8-140 21067609 PMC2992540

[B25] Barad D Brill H Gleicher N Update on the use of dehydroepiandrosterone supplementation among women with diminished ovarian function J Assist Reprod Genet 2007 24 629 634 10.1007/s10815-007-9178-x 18071895 PMC3454995

[B26] Gleicher N Ryan E Weghofer A Blanco-Mejia S Barad DH Miscarriage rates after dehydroepiandrosterone (DHEA) supplementation in women with diminished ovarian reserve: a case control study Reprod Biol Endocrinol 2009 7 108 10.1186/1477-7827-7-108 19811650 PMC2764711

[B27] Gleicher N Weghofer A Barad DH Improvement in diminished ovarian reserve after dehydroepiandrosterone supplementation Reprod Biomed Online 2010 21 360 365 10.1016/j.rbmo.2010.04.006 20638339

[B28] Sönmezer M Ozmen B Cil AP Ozkavukçu S Taşçi T Olmuş H Atabekoğlu CS Dehydroepiandrosterone supplementation improves ovarian response and cycle outcome in poor responders Reprod Biomed Online 2009 19 508 513 10.1016/j.rbmo.2009.06.006 19909591

[B29] Weissman A Horowitz E Ravhon A Golan A Levran D Dehydroepiandrosterone supplementation increases baseline follicular phase progesterone levels Gynecol Endocrinol 2011 27 1014 1017 10.3109/09513590.2011.569611 21500990

[B30] Hyman JH Margalioth EJ Rabinowitz R Tsafrir A Gal M Alerhand S Algur N Eldar-Geva T DHEA supplementation may improve IVF outcome in poor responders: a proposed mechanism Eur J Obstet Gynecol Reprod Biol 2013 168 49 53 10.1016/j.ejogrb.2012.12.017 23312476

[B31] Gleicher N Kim A Weghofer A Shohat-Tal A Lazzaroni E Lee HJ Barad DH Starting and resulting testosterone levels after androgen supplementation determine at all ages in vitro fertilization (IVF) pregnancy rates in women with diminished ovarian reserve (DOR) J Assist Reprod Genet 2013 30 49 62 10.1007/s10815-012-9890-z 23212832 PMC3553353

[B32] Hyman JH Margalioth EJ Rabinowitz R Tsafrir A Algur N Eldar-Geva T Dehydroepiandrosterone (DHEA) supplementation for poor responders - How does it work? Fertil Steril 2010 94 S86

[B33] Gleicher N Goyal A Weghofer A Barad D Supplementation with dehydroepiandrosterone (DHEA) improves ovarian reserve, as reflected by anti-mullerian hormone levels Fertil Steril 2009 92 S54 S55

[B34] Goyal A Barad D Weghofer A Oktay K Gleicher N Predicting improvements in ovarian reserve and pregnancy rates after supplementation of dehydroepiandrosterone (DHEA) in diminished ovarian reserve Hum Reprod 2009 24 i79

[B35] Gleicher N Barad DH Dehydroepiandrosterone (DHEA) supplementation in diminished ovarian reserve (DOR) Reprod Biol Endocrinol 2011 9 67 10.1186/1477-7827-9-67 21586137 PMC3112409

[B36] Yakin K Urman B DHEA as a miracle drug in the treatment of poor responders; hype or hope? Hum Reprod 2011 26 1941 1944 10.1093/humrep/der150 21593043

[B37] Urman B Yakin K Does dehydroepiandrosterone have any benefit in fertility treatment? Curr Opin Obstet Gynecol 2012 24 132 135 10.1097/GCO.0b013e32835175c3 22327737

[B38] Sunkara SK Coomarasamy A Arlt W Bhattacharya S Should androgen supplementation be used for poor ovarian response in IVF? Hum Reprod 2012 27 637 640 10.1093/humrep/der464 22252080

[B39] Kolibianakis EM Venetis CA Tarlatzis BC DHEA administration in poor responders Hum Reprod 2011 26 730 731 author reply 731 10.1093/humrep/deq397 21242147

[B40] Ferraretti AP La Marca A Fauser BC Tarlatzis B Nargund G Gianaroli L Definition EwgoPOR: ESHRE consensus on the definition of 'poor response' to ovarian stimulation for in vitro fertilization: the Bologna criteria Hum Reprod 2011 26 1616 1624 10.1093/humrep/der092 21505041

[B41] Broekmans FJ Soules MR Fauser BC Ovarian aging: mechanisms and clinical consequences Endocr Rev 2009 30 465 493 10.1210/er.2009-0006 19589949

[B42] Bosdou JK Venetis CA Kolibianakis EM Toulis KA Goulis DG Zepiridis L Tarlatzis BC The use of androgens or androgen-modulating agents in poor responders undergoing in vitro fertilization: a systematic review and meta-analysis Hum Reprod Update 2012 18 127 145 10.1093/humupd/dmr051 22307331

